# Multilevel modeling for the analysis of longitudinal blood pressure data in the Framingham Heart Study pedigrees

**DOI:** 10.1186/1471-2156-4-S1-S19

**Published:** 2003-12-31

**Authors:** Laurent Briollais, Anjela Tzontcheva, Shelley Bull

**Affiliations:** 1 Division of Epidemiology and Biostatistics, Samuel Lunenfeld Research Institute, Mount Sinai Hospital, 600 University Avenue, Toronto, Ontario, Canada, M5G 1X5; 2 Department of Public Health Sciences, University of Toronto, Toronto, Ontario, Canada, M5G 1X5

## Abstract

**Background:**

The data arising from a longitudinal familial study have a complex correlation structure that cannot be modeled using classical methods for the analysis of familial data at a single time point.

**Methods:**

To fit the longitudinal systolic blood pressure (SBP) pedigree data arising from the Framingham Heart Study, we proposed to use multilevel modeling. That approach was used to distinguish multiple levels of information with individual repeated measurements (Level 1) being made within individuals (Level 2), and individuals clustered within pedigrees (Level 3). Residuals from the subject-specific and pedigree-specific regression models were summed both for the mean SBP and slope of SBP change over time, in order to define two new outcomes that were then used in a genome-wide linkage analysis.

**Results:**

Evidence for linkage for the two outcomes (mean SBP and slope) was found in several chromosomal regions with a maximum LOD score of 3.6 on chromosome 8 and 3.5 on chromosome 17 for the mean SBP, and 2.5 on chromosome 1 for SBP slope. However, the linkage on chromosome 8 was only detected when the sample was restricted to subjects between age 25 and 75 and with at least four exams (Cohort 1) or 3 exams (Cohort 2).

**Discussion:**

Multilevel modeling is a powerful approach to detect genes involved in complex traits when longitudinal data are available. It allows for complex hierarchical data structure to be taken into account and therefore, a better partitioning of random within-individual variation from other sources of variability (genetic or nongenetic).

## Background

The Framingham Heart Study provides long-term repeated measurements of blood pressure and other phenotypes in two large cohorts of related individuals. Longitudinal studies are efficient designs for the investigation of individual changes over time. In the context of familial studies, such designs might be of particular interest to assess the proportion of the trait variability explained by within-individual variation or other sources of variation. However, the data arising from a longitudinal familial study have a complex correlation structure that cannot be modeled using classical methods for the analysis of familial data at a single time point. In this study, we proposed to use multilevel modeling to fit the complex data structure arising from the Framingham Heart Study. Multilevel modeling, also known as hierarchical regression, generalizes ordinary regression modeling to distinguish multiple levels of information in a model [1]. It might be appropriate to model the Framingham Heart Study data that form a natural hierarchy with individual repeated measurements (Level 1) being made within individuals (Level 2), and individuals clustered within pedigrees (Level 3). The use of appropriate random effects at each level allows one to adjust for the influence of a wide variety of correlation structures and to estimate variance, covariance, and correlation which are of particular interest in familial studies. In this paper, multilevel models are first used to fit the repeated systolic blood pressure (SBP) measurements. Residuals from the subject-specific and pedigree-specific regression models were summed both for the mean SBP and slope of SBP change over time, in order to define two new outcomes that were then used in a genome-wide linkage analysis. Both phenotypes are of interest because genes involved in the variation of SBP with time could differ from genes affecting long-term mean SBP.

## Methods

### Data

The Framingham Heart Study data includes 330 pedigrees originally selected for a genome-scan analysis. The pedigrees consisted of 4692 subjects, of whom 2885 have participated in the Framingham Heart Study. Longitudinal SBP data were analyzed for 25,263 examinations on 2662 individuals. Height, weight, gender, age, and hypertensive treatment information were required but if height was missing, the most recent measurement was imputed. Because there might be important variation in individual SBP measurement among younger and older subjects, we also restricted the sample to individuals aged between 25 and 75 years, as in Levy et al. [2]. The following selection criteria were also defined: 1) There had to be at least 10 years between a subject's initial and final examinations within the age range; 2) at least four examinations within the age range were required for the original cohort and at least three for offspring cohort participants [2]. Data from 24,840 examinations on 2530 individuals were available in the selected sample. For the genome-wide scan analysis, 1702 genotyped individuals were included (394 from the Cohort 1 and 1308 from the Cohort 2).

### Multilevel analysis of the longitudinal SBP model

Let the random variable *Y*_*ijk *_denote the SBP measurement at the *i*^th ^examination for the *j*^th ^individual in pedigree *k*. We then assume that *Y*_*ijk *_satisfies the following general multilevel model:

#### Within-subject model – Level 1



where *i *= 1,...,21 for Cohort 1 subjects and *i *= {11, 15, 17, 19, 21} for Cohort 2 subjects. *Age*_*ijk*_, *BMI*_*ijk*_, *Treat*_*ijk *_are the age, body mass index and hypertension treatment (1 for subjects treated and 0 for subjects untreated) at the *i*^th ^exam for the *j*^th ^individual in pedigree *k*,  and  are the mean values across all exams for the *j*^th ^individual, and ε_*ijk *_are the error components that account for the within-individual variability. The ε_*ijk *_are assumed to be normally distributed with mean vector zero and variance-covariance matrix Σ defined by a first-order autoregressive structure. The intercept *b*_0*jk *_represents the average SBP for an untreated subject of average age and BMI across all of the subject's examinations. The regression coefficient *b*_1*jk *_is used to model the linear variation of SBP with age. We found that every individual profile could be well approximated by a quadratic function of time, measured by the age at examination. We also tested a cubic effect, but it was not significant when we allowed for the individual's linear time trend to differ in each treatment group (interaction between age and treatment). Random effects were added to reflect the natural heterogeneity in the population. In this model, both the intercept and the linear effect for age were allowed to vary across individuals and the individual-specific regression coefficients (random effects) were defined at the second level:

#### Subject random-intercept model – Level 2



#### Subject random-slope model – Level 2



 and  are the sample means for age and body mass index, *Sex *and *Cohort *are two indicator variables, coded 1 for males, 0 for females and 0 for Cohort 1 subjects, 1 for Cohort 2 subjects. The random components *u*_0*jk *_and *u*_1*jk *_measure the variation of each individual's mean SBP and slope from their average in pedigree *k*. The intercept *b*_00*k *_represents the average SBP in pedigree *k *for males in Cohort 1 with average age and BMI and the intercept *b*_10*k *_represents the average slope in pedigree *k *for males in Cohort 1 with average BMI. To account for the correlation of individuals within a pedigree, these two intercepts were allowed to vary between pedigrees. The random effects at different levels of the model are assumed independent.

#### Pedigree random-intercept model – Level 3

*b*_00*k *_= β_000 _+ *v*_00*k*_, *k *= 1,...,*N*

#### Pedigree random-slope model – Level 3



The random components *v*_00*k *_and *v*_01*k *_measure the variation of each pedigree's mean SBP and mean slope from their average in the whole sample.

### Statistical tests in the multilevel model

Analyses were conducted in both the unselected and selected samples and with and without adjustment for BMI. Multilevel models were fitted using SAS PROC MIXED [3]. Parameter estimates are obtained by restricted maximum likelihood estimation (REML). An *F*-statistic was used to test the significance of the fixed effects with number of degrees of freedom computed using the containment method [4]. The likelihood ratio statistic based on REML likelihoods was used to test the significance of the random effects. The null distribution of this statistic is a mixture of  and  with equal weights 0.5, where *q *and *q *+ 1 are the number of random effects estimated under *H*_0 _and *H*_1_, respectively.

### Genome-wide linkage analysis

We used the estimates of the random effects at the subject and pedigree levels to define two new outcomes that were used in the genome-wide linkage analysis. The two outcomes were defined as  and , which measure the random variation of each individual's SBP mean and slope, respectively, from the sample average after adjustment for the fixed effects. A third outcome was also defined using the residuals from a sample-wide regression in which each individual's mean SBP (across all exams) was regressed on his mean age (centered), mean BMI (centered), gender and cohort, as in Levy et al.'s paper [2]. Estimation of heritability and two-point linkage analyses were performed on the pedigree data using the variance component models implemented in the SOLAR package [5].

## Results

### Multivariate analysis of longitudinal SBP

**Figure 1:**
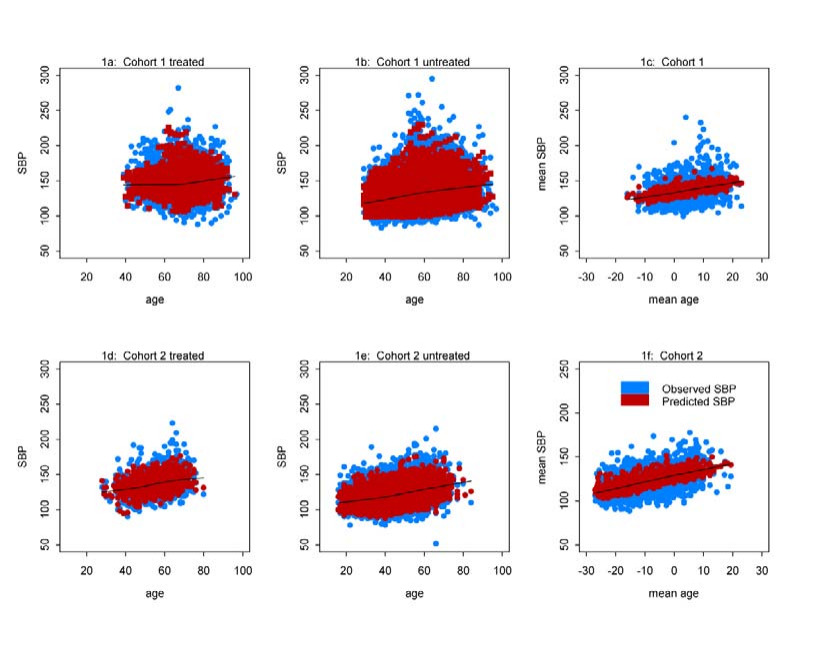
**Comparisons of observed SBP and predicted values for the selected sample **A, B, D, E are based on the multilevel model. C and F are based on the sample-wide regression for the selected sample [2].

All fixed effects included in the model were highly significant in the subject random slope model (Table 1) except for gender. Most of the SBP variability (316.8 in Model 1a, Table 1) was explained by within-subject (140.8, 44%) and between-subject (146.2, 46%) variability in the mean SBP and to a lesser extent by between-pedigree variability (27.6, <9%). Much less variability was explained by variability in the slope (0.17+0.008, <0.06%). Pedigree effects of mean SBP and SBP slope were more significant when the multilevel analyses were adjusted for body mass index. As shown in Figure 1, the multilevel model fit well the data while the sample-wide regression does not capture all the SBP variability.

### Heritability

Heritability estimates were 54.3% (SE = 3.1) and 55.6% (SE = 3.4) for the mean SBP, 31.9% (SE = 3.5) and 28.9% (SE = 3.5) for SBP slope over time in the unselected and selected samples, respectively. The heritability estimates for the subject-specific residuals from the sample-wide regression of the mean SBP were 47.7% (SE = 3.4) and 49.7% (SE = 3.8) in the unselected and selected samples, respectively.

### Genome-wide linkage analysis

**Figure 2:**
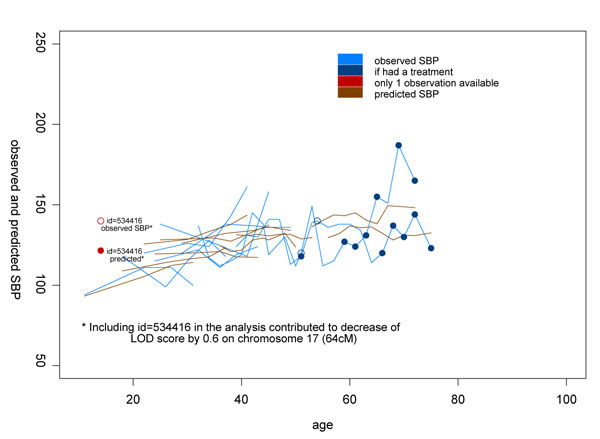
Observed and predicted SBP profiles for pedigree 32,445 members

Evidence for linkage for the two outcomes (mean SBP and slope) was found in several chromosomal regions with a maximum LOD score of 3.6 on chromosome 8 and 3.5 on chromosome 17 for the mean SBP and 2.5 on chromosome 1 for SBP slope (Table 2). However, linkage on chromosome 8 for the mean SBP was only found in the selected sample. The decrease in LOD score in the unselected sample on chromosome 17 was important in several pedigrees that included individuals with a single SBP measurement, as illustrated in Figure 2. Adjusting the analyses for BMI showed stronger evidence for linkage, which could suggest that BMI is determined by other genetic factors (Table 3). Not adjusting the analysis for treatment effect did not change the results of the mean SBP, but yielded lower LOD scores for SBP slope (Table 3).

## Discussion

Our study demonstrates the value of multilevel modeling in the search for genetic determinants of complex traits when longitudinal pedigree data are available. For the mean SBP, we were able to replicate the linkage result on chromosome 17 previously reported by Levy et al. [2] and detect a new linkage on chromosome 8 that was not reported before. For SBP slope, we also found suggestive results for linkage for both mean SBP and SBP slope on several other chromosomal regions, including chromosomes 1, 2, 3, 11, and 13. Using residuals from the multilevel model in a genome-wide linkage analysis gave stronger evidence for linkage than using residuals from a sample-wide regression as in the Levy et al.'s paper [2]. This might be because this latter approach does not correctly account for within-individual and between-individual variability. Multilevel modeling, which can take into account the hierarchical structure of the data, may help disentangle the proportion of the trait variability explained by fundamental variation in the mean SBP and in the SBP slope from the proportion explained by random within-individual variability. A more general hierarchical structure could have included a nuclear family level nested within the pedigree level. However, such a multilevel model would be more difficult to fit. In our analysis we only included a fixed cohort effect that could account for differences between generations within a pedigree. Treating the pedigrees as random effects also allowed for between-pedigree heterogeneity in our model, which improved the accuracy of the random effect estimates at the individual level. Although there may be some concern about using a two-stage approach for detecting linkage, other studies based on similar strategies using linear mixed models in simulated data did not report an inflation of type I error for the test of linkage in the context genome-wide linkage analysis [6,7]. The linkage on chromosome 17 for mean SBP was only found in the selected sample. A important decrease in LOD score (>0.1) in the unselected sample was observed in several pedigrees comprising individuals with a single extreme SBP measurement, as illustrated in Figure 2. This suggests that a single SBP measurement may not provide a reliable characterization for an individual, especially when a familial study of SBP is designed. Adjusting the analyses for BMI showed stronger evidence for linkage, which could suggest that BMI is determined by other genetic factors. No correction was applied to the SBP value of subjects who received a hypertensive treatment. The analyses with the multilevel model were adjusted for treatment effect so that the residuals obtained from this model correspond to the untreated group. Taking into account an interaction between age and treatment in the multilevel model may also have reduced the bias due to treatment effect. However, our linkage results were insensitive to whether the analyses were adjusted for treatment effect. The multilevel modeling approach is also known to be robust to missing data, under the assumption that they are missing at random [4]. Future work could include the development of an integrated approach to perform linkage analysis within the multilevel framework.

## Figures and Tables

**Table 1:** Estimates of multilevel model fixed effects and random effects variances (± SE) in the selected and unselected samples with or without adjustment for BMI

	**Unselected Sample**		**Selected Sample**	
	
	**Adjusted (1a)**	**Unadjusted (1b)**	**Adjusted (2a)**	**Unadjusted (2b)**
***Fixed effects estimates***				
Intercept	132.46 (0.56)***	132.00 (0.57)***	131.16 (0.58)***	131.80 (0.59)***
Age effect^A^	0.60 (0.02)***	0.63 (0.02)***	0.61 (0.02)***	0.63 (0.02)***
Age^2 ^effect^A^	0.013 (0.001)***	0.010 (0.001)***	0.014 (0.001)***	0.001 (0.001)***
BMI effect^A^	1.45 (0.05)***	-	1.4 (0.05)***	-
Treat effect	-2.13 (0.42)***	-1.45 (0.43)***	-2.2 (0.43)***	-1.52 (0.44)***
Age * Treatment effect	-0.35 (0.04)***	-0.34 (0.04)***	-0.34 (0.04)***	-0.34 (0.04)***
*Subject random intercept model*				
Mean age effect^A^	0.53 (0.03)***	0.65 (0.03)***	0.62 (0.04)***	0.72 (0.04)***
Mean BMI effect^A^	1.16 (0.06)***	-	1.21 (0.07)***	-
Cohort effect	-6.56 (0.67)***	-5.21 (0.70)***	-5.74 (0.71)***	-4.68 (0.74)***
Gender effect	2.27 (0.51)***	3.59 (0.53)***	1.71 (0.53)***	3.09 (0.56)***
*Subject random slope model*				
Mean BMI effect^A^	-0.014 (0.003)***	-	-0.015 (0.003)***	-
Cohort effect	-0.46 (0.03)***	-0.29 (0.03)***	-0.42 (0.03)***	-0.27 (0.03)***
Gender effect	-0.03 (0.03)	-0.07 (0.03)*	-0.04 (0.03)	-0.08 (0.03)**
***Covariance Parameter Estimates***				
Measurement error variance	140.85 (1.64)***	148.11 (1.73)***	141.80 (1.68)***	149.01 (1.77)***
AR(1) correlation	0.25 (0.01)***	0.26 (0.01)***	0.25 (0.01)***	0.26 (0.01)***
*Subject level *var(u_0jk_)	146.25 (5.01)***	161.79 (5.49)***	147.63 (5.30)***	164.31 (5.84)***
var(u_1jk_)	0.17 (0.01)***	0.20 (0.01)***	0.17 (0.01)***	0.20 (0.01)***
cov(u_0jk_, u_1jk_)	1.41 (0.19)***	0.85 (0.21)***	1.42 (0.20)***	0.86 (0.22)***
*Pedigree level *var(v_00k_)	27.59 (4.17)***	24.41 (4.07)***	27.04 (4.31)***	23.22 (4.13)***
var(v_01k_)	0.008 (0.005)*	0.005 (0.005)	0.009 (0.005)*	0.006 (0.005)
cov(v_00k_, v_01k_)	0.52 (0.12)***	0.37 (0.11)**	0.49 (0.12)***	0.37 (0.12)**
**-2 REML**	202,695.75	205,876.11	194,947.18	198,006.24

^A^Variables centered * *p *< 0.05 ** *p *< 0.01 *** *p *< 0.001

**Table 2:** Results of two-point linkage analysis (LOD scores ≥ 2 are in bold)

		**Unselected Sample**			**Selected Sample**		
		
Markers	Location	Mean SBP Multilevel Model	Mean SBP^A ^ Sample-wide Regression	SBP slope Multilevel Model	Mean SBP Multilevel Model	Mean SBP^A ^ Sample-wide Regression	SBP slope Multilevel Model
GATA48B01	1 (212 cM)	0.30	0.23	**2.00**	0.30	0.21	**2.47**
GATA11H10	2 (38 cM)	1.47	1.36	0	**2.32**	**2.20**	0.18
GATA6F06	3 (79cM)	**2.16**	1.78	0	1.17	0.81	0.14
GATA4A10	3 (153 cM)	0.31	0.21	**2.27**	0.33	0.31	1.29
GATA72C10	8 (37 cM)	**3.57**	**2.54**	1.00	**3.57**	**2.82**	1.71
ATA34E08	11 (33 cM)	0.16	0.11	**2.13**	0.03	0.01	1.54
GATA7G10	13 (64 cM)	**2.02**	1.92	0	1.79	1.63	0
GATA25A04	17 (62 cM)	**2.13**	1.78	0	**3.54**	**3.42**	0

^A^Residuals from the sample-wide regression of the mean SBP on the mean age, mean BMI, gender, and cohort effects as in Levy et al. [2].

**Table 3:** Results of two-point linkage analysis in the selected sample using residuals from the multilevel model (LOD scores ≥ 2 are in bold)

		**Mean SBP** ** Analysis adjusted for^A^**			**SBP Slope** ** Analysis adjusted for^A^**		
		
**Markers**	**Location**	BMI, treatment	BMI only	Treatment only	BMI, treatment	BMI only	Treatment only
GATA48B01	1 (212 cM)	0.30	0.30	0.17	**2.47**	1.83	1.53
GATA11H10	2 (38 cM)	**2.32**	**2.53**	0.93	0.18	0.25	0
GATA6F06	3 (79 cM)	1.17	1.21	1.49	0.14	0.02	0.06
GATA4A10	3 (153 cM)	0.33	0.32	0.02	1.29	0.74	0.70
GATA72C10	8 (37 cM)	**3.57**	**3.52**	1.32	1.71	1.13	0.68
ATA34E08	11 (33 cM)	0.03	0.02	0.01	1.54	1.19	0.93
GATA7G10	13 (64 cM)	1.79	1.68	0.71	0	0	0
GATA25A04	17 (62 cM)	**3.54**	**3.36**	1.48	0	0	0

^A^All analyses are also adjusted for age effect in Level 1 and age, sex, and Cohort effects in Level 2.
